# Identification of key genes for atherosclerosis in different arterial beds

**DOI:** 10.1038/s41598-024-55575-8

**Published:** 2024-03-19

**Authors:** Xize Wu, Xue Pan, Yi Zhou, Jiaxiang Pan, Jian Kang, J. J. Jiajia Yu, Yingyue Cao, Chao Quan, Lihong Gong, Yue Li

**Affiliations:** 1 Nantong Hospital of Traditional Chinese Medicine, Nantong Hospital Affiliated to Nanjing University of Chinese Medicine, No. 41 Jianshe Road, Chongchuan District, Nantong, 226000 Jiangsu China; 2 Liaoning University of Traditional Chinese Medicine, Shenyang, 110847 Liaoning China; 3 The Affiliated Hospital of Liaoning University of Traditional Chinese Medicine, No. 33, Beiling Street, Huanggu District, Shenyang, 110032 Liaoning China; 4 Liaoning Provincial Key Laboratory of TCM Geriatric Cardio-Cerebrovascular Diseases, Shenyang, 110847 Liaoning China; 5 Dazhou Vocational College of Chinese Medicine, Dazhou, 635000 Sichuan China

**Keywords:** Atherosclerosis, Macrophage polarization, Unsupervised clustering analysis, LASSO regression, SVM-REF, Diagnostic markers, Computational biology and bioinformatics

## Abstract

Atherosclerosis (AS) is the pathologic basis of various cardiovascular and cerebrovascular events, with a high degree of heterogeneity among different arterial beds. However, mechanistic differences between arterial beds remain unexplored. The aim of this study was to explore key genes and potential mechanistic differences between AS in different arterial beds through bioinformatics analysis. Carotid atherosclerosis (CAS), femoral atherosclerosis (FAS), infrapopliteal atherosclerosis (IPAS), abdominal aortic atherosclerosis (AAS), and AS-specific differentially expressed genes (DEGs) were screened from the GSE100927 and GSE57691 datasets. Immune infiltration analysis was used to identify AS immune cell infiltration differences. Unsupervised cluster analysis of AS samples from different regions based on macrophage polarization gene expression profiles. Weighted gene co-expression network analysis (WGCNA) was performed to identify the most relevant module genes with AS. Hub genes were then screened by LASSO regression, SVM-REF, and single-gene differential analysis, and a nomogram was constructed to predict the risk of AS development. The results showed that differential expression analysis identified 5, 4, 121, and 62 CAS, FAS, IPAS, AAS-specific DEGs, and 42 AS-common DEGs, respectively. Immune infiltration analysis demonstrated that the degree of macrophage and mast cell enrichment differed significantly in different regions of AS. The CAS, FAS, IPAS, and AAS could be distinguished into two different biologically functional and stable molecular clusters based on macrophage polarization gene expression profiles, especially for cardiomyopathy and glycolipid metabolic processes. Hub genes for 6 AS (ADAP2, CSF3R, FABP5, ITGAX, MYOC, and SPP1), 4 IPAS (CLECL1, DIO2, F2RL2, and GUCY1A2), and 3 AAS (RPL21, RPL26, and RPL10A) were obtained based on module gene, gender stratification, machine learning algorithms, and single-gene difference analysis, respectively, and these genes were effective in differentiating between different regions of AS. This study demonstrates that there are similarities and heterogeneities in the pathogenesis of AS between different arterial beds.

## Introduction

Atherosclerosis (AS) is a common clinical vascular pathology, which starts from the intima and is characterized by localized lipid accumulation, fibrous tissue proliferation, and calcium deposits, and ultimately plaque formation. The development of AS primarily occurs in large and medium-sized arteries, such as carotid arteries, femoral arteries, and abdominal aorta^1^. However, studies have indicated that deceased patients without cardiovascular events are more susceptible to AS in the abdominal aorta^2^; smoking accelerated AS and plaque formation in the abdominal aorta in adolescents without affecting right coronary artery^3^; diabetes significantly accelerated plaque formation in the lower limb arteries, and hypertension appeared to occur preferentially occur in the carotid arteries^4^; and superficial femoral atherosclerosis develops more slowly than coronary and carotid arteries^5^. In addition, carotid atherosclerosis (CAS) is more likely to cause ischemic cerebrovascular events; coronary atherosclerosis is associated with an increased risk of ischemic heart disease; lower extremity atherosclerosis tends to result in lower extremity arterial occlusion; whereas abdominal aortic atherosclerosis (AAS) seems to contribute to abdominal aortic aneurysms as well as renal and lower extremity blood supply deficiencies. These findings suggest significant differences in the progression of AS between individuals, with different arterial beds exhibiting heterogeneity^6,7^. The formation of AS involves the mechanisms of lipid deposition, inflammation, and immune response. Nevertheless, femoral atherosclerosis (FAS) shows higher levels of ossification and calcification but lower cholesterol concentrations than carotid atherosclerosis^8^. Superficial femoral artery plaques have a more fibrous component and less lipid compared to coronary arteries^9^. This also demonstrates the heterogeneity of AS mechanisms between different arterial beds.

To better understand the expression differences of AS in various arterial beds, this study focused on CAS, FAS, infrapopliteal atherosclerosis (IPAS), and AAS. First, differential expression analysis was conducted to identify genes that distinguish the AS groups from normal samples. Subsequently, differential expression analysis was performed to identify genes that differentiate AS in different arterial beds. Finally, machine learning and single-gene differential analysis were employed to screen for hub genes, and a nomogram was constructed to predict the risk of morbidity.

## Materials and methods

### Subjects and dataset acquisition

**Figure 1:**
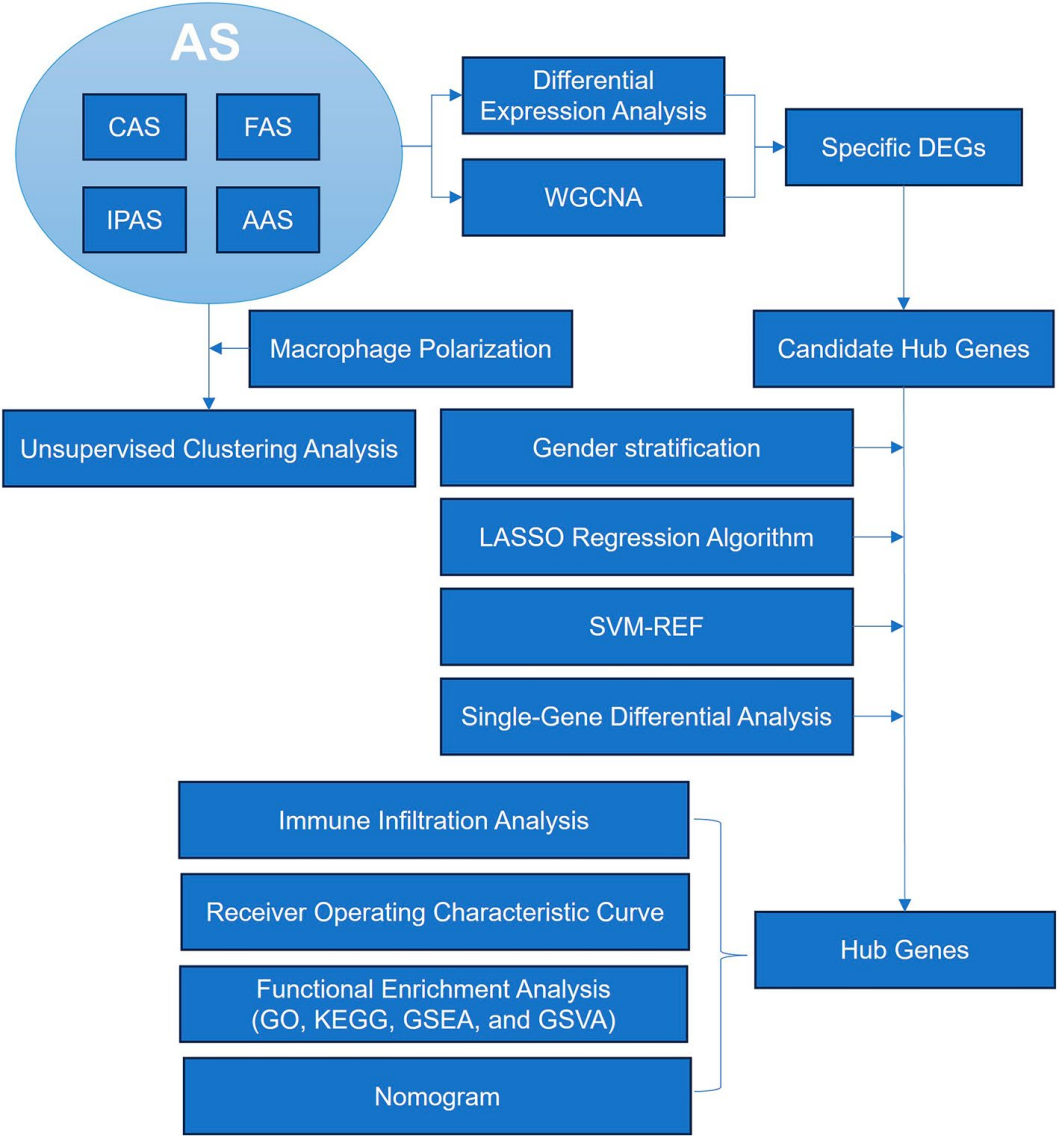
Flow chart of this study.

The entire study process is depicted in Fig. 1. Two gene expression profiles (GSE100927^10^ and GSE57691^11^) were retrieved from the GEO database (https://www.ncbi.nlm.nih.gov/geo/). Among them, GSE100927 included 12 carotid normal and 29 CAS tissues; 12 femoral normal and 26 FAS tissues; 11 infrapopliteal normal and 14 IPAS tissues; and GSE57691 included 10 abdominal aortic normal and 9 AAS tissues.

### Identification of differentially expressed genes (DEGs)

The GSE100927 and GSE57691 datasets were merged and standardized using the “Affy” R package^12^, while batch effects were removed with the “SVA” R package^13^. DEGs were identified by comparing disease and control groups using the “limma” R package, with the criteria set at *P* < 0.05 and |logFC| > 1.0 for DEG selection^14^. Macrophage polarization-related genes were obtained from the GeneCards (http://www.genecards.org/) database with a “relevance score” > 10^15^.

### Gene Set Enrichment Analysis (GSEA)

The “GSEA” package was used to explore the related pathways of specific genes and to calculate the correlation between specific genes and other genes in R. All genes were then sorted from highest to lowest according to their correlation, and these sorted genes were the set of genes to be tested. The KEGG signaling pathway set was called a “predefined set” to detect its enrichment in the gene set.

### Gene Ontology (GO) and Kyoto Encyclopedia of Genes and Genomes (KEGG) enrichment analysis

Genes were imported into the David database (https://david.abcc.ncifcrf.gov/) for GO and KEGG analysis, including biological processes, cellular components, molecular functions, and signaling pathways, and the species was limited to “Homo sapiens,” with a *P* < 0.05 as the screening condition^16,17^.

### Immune infiltration analysis and correlation analysis

The degree of infiltration of 22 immune cells was quantified using the CIBERSORT deconvolution algorithm based on gene microarray data^18^. CIBERSORTx (https://cibersortx.stanford.edu/) was used to analyze immune cell infiltration for each sample^19^. Differences between the two groups were compared using the Wilcox test.

### Unsupervised clustering analysis of AS samples from different regions

Unsupervised clustering analysis of AS samples based on macrophage polarization expression profiles was performed using the “ConsensusClusterPlus” R package^20^. The AS samples from different regions were grouped by applying the k-means algorithm with 1000 iterations and k = 9. The appropriate number of clusters was determined based on the cumulative distribution function curve, consensus matrix, and consistent cluster score (> 0.9)^20^.

### Gene Set Variation Analysis (GSVA)

The “GSVA” package was used to conduct a GSVA enrichment analysis for different clusters, considering a significant change if the |t value of the GSVA score| was greater than two^21^.

### Weighted Gene Co-expression Network Analysis (WGCNA)

WGCNA was employed to identify co-expression modules by clustering the samples using the “WGCNA” R package^22^. Outlier samples were removed, and a co-expression network for the gene expression matrices of the remaining samples was constructed. Appropriate soft thresholds were determined to identify gene modules and select the most relevant ones.

### Machine learning models screening for Hub genes

The candidate Hub genes were screened with the Support Vector Machine-Recursive Feature Elimination (SVM-RFE) algorithm and Least Absolute Shrinkage and Selection Operator Regression (LASSO) regression analysis. The LASSO algorithm was used to select the variables and adjust the complexity^23^. The SVM-RFE algorithm analyzes the most appropriate key genes^24^.

### Single-gene differential analysis

Hub genes were identified by comparing the expression differences of candidate Hub genes in different groups by single gene differential analysis. The one-way analysis of variance (ANOVA) was performed between multiple groups using the “car” R package. One-way ANOVA was used for normal distribution with homogeneous variance, Welch's ANOVA for heterogeneous variance, and Bonferroni's method was used to correct for pairwise comparisons. The Kruskal-Walli’s test was used for non-normal distributions, and the Wilcox rank sum test was used for pairwise comparisons.

### Construction and validation of a nomogram model

A nomogram model was established using the “rms” R package to predict the probability of the occurrence of AS in different regions, and its predictive power was estimated by using calibration curves and decision curve analysis. The area under the curve (AUC) of the subject operating characteristic (ROC) was then calculated for each key gene to test the diagnostic efficacy of the Hub genes.

### Statistical analysis

All statistical analyses were performed using R software, and *P* < 0.05 was considered significant.

## Results

### Identification of DEGs

**Figure 2:**
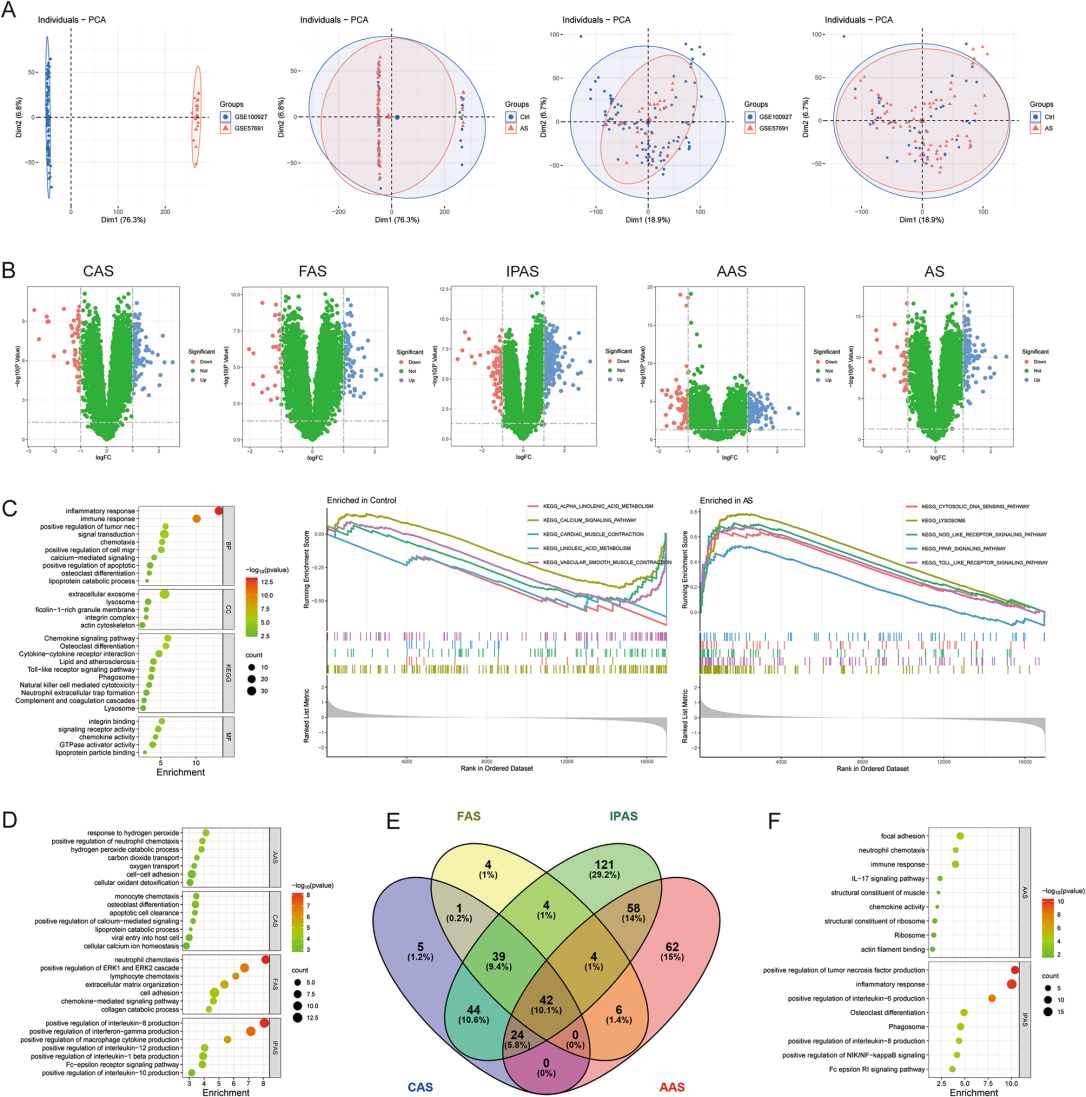
Identification and functional enrichment analysis of differentially expressed genes (DEGs). (**A**) The principal component analysis of the two datasets and clinical characteristics; (**B**) The volcano plot of DEGs for CAS, FAS, IPAS, AAS, and AS; (**C**-**D**) The functional enrichment analysis and GEEA analysis of AS; (**E**) Venn diagram showing specific DEGs; (**F**) The functional enrichment analysis of AAS- and IPAS-specific DEGs.

The differential expression analysis of CAS, FAS, IPAS, AAS, and AS identified 229, 104, 35, 119, and 159 DEGs, respectively (Fig. 2A,B). Firstly, the relevant pathogenesis of AS was explored, and functional enrichment analysis showed that its pathogenesis is closely related to inflammatory response, cell migration and chemotaxis (chemotaxis of neutrophils, monocytes, lymphocytes, and macrophages), cell death, lipid metabolism, and immune response (Fig. 2C). Then, enrichment analysis showed that CAS was associated with cell differentiation (osteoclast, dendritic, and myeloid cell differentiation), ion transfer (membrane repolarization during atrial cardiac muscle cell action potential, potassium ion transport, regulation of cellular calcium homeostasis); FAS was enriched in cell chemotaxis-related pathways; IPAS was involved in the regulation of various interleukin factors (IL-10, IL-12, IL-1β, IL-8); and AAS was mainly involved in cellular redox reactions (Fig. 2D). In order to explore the potential specific pathogenesis of AS in different regions, 5 CAS-specific DEGs, 4 FAS-specific DEGs, 121 IPAS-specific DEGs, 62 AAS-specific DEGs, and 42 common DEGs for AS were obtained by Venn diagram, respectively (Fig. 2E). The enrichment analysis of these genes showed that IPAS is closely associated with interleukin factor regulation and osteoclast differentiation; AAS is closely related to inflammatory response (IL-17 signaling pathway, chemokine signaling pathway) and cell migration (neutrophil chemotaxis, focal adhesion, actin filament binding) (Fig. 2F).

**Figure 3:**
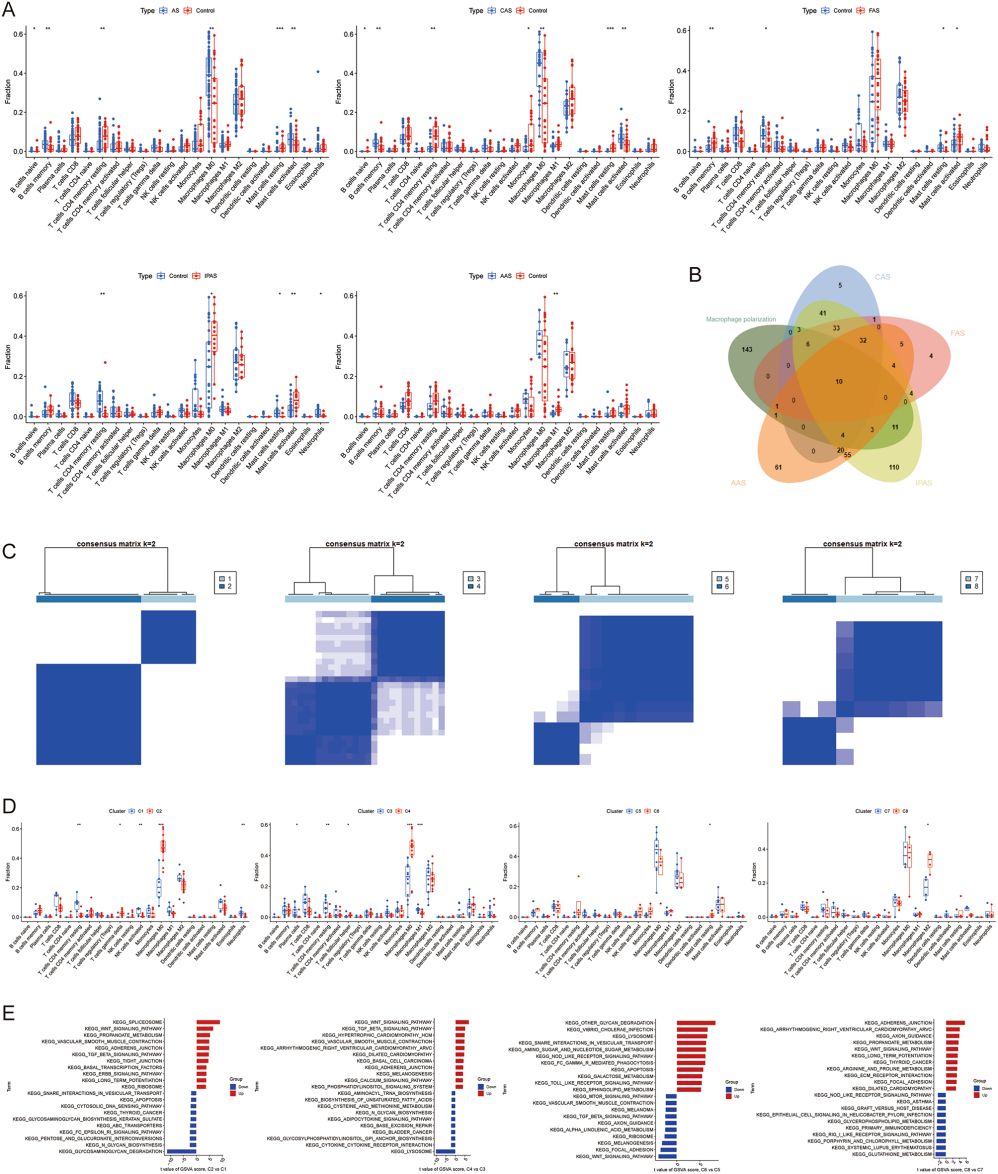
Identification of macrophage polarization-related molecular clusters. (**A**) Immune infiltration analysis of AS, CAS, FAS, IPAS, and AAS; (**B**) Venn diagram showing macrophage polarization and AS intersection genes; (**C**) Consensus clustering matrix when k = 2; (**D**) Immune infiltration analysis of different molecular clusters; (**E**) The GSVA analysis of different molecular clusters. *P < 0.05, **P < 0.001, ***P < 0.0001.

To further understand the immune microenvironment of AS in different regions, the results showed that AS had a higher abundance of memory B cells, M0 macrophages, and activated mast cells, and lower abundance of naive B cells, resting memory CD4 T cells, and resting mast cells (*P* < 0.05); CAS had a higher abundance of memory B cells, M0 macrophages, and activated mast cells, and lower abundance of naive B cells, resting memory CD4 T cells, monocytes, and resting mast cells (*P* < 0.05); FAS had a higher abundance of memory B cells and activated mast cells, and lower abundance of resting memory CD4 T cells and resting mast cells (*P* < 0.05); IPAS had a higher abundance of M0 macrophages and activated mast cells, had lower abundance of resting memory CD4 T cells, resting mast cells, and neutrophils (*P* < 0.05); and AAS had a lower abundance of M1 macrophages (*P* < 0.05) (Fig. 3A). It is apparent that macrophages and mast cells are differently enriched in different regions of the AS, and we believe that macrophage polarization plays an important role.

### Identification of macrophage polarization molecular clusters

The GeneCards database was searched to obtain 182 macrophage polarization-related genes with a “relevance score” > 10, and 23, 17, 37, and 19 intersected genes were obtained by intersecting with the specific DEGs of CAS, FAS, IPAS, and AAS, respectively. To explore the role of macrophage polarization on AS in different regions, unsupervised cluster analysis was performed on 29 CAS samples, 26 FAS samples, 14 IPAS samples, and 9 AAS samples based on the expression profiles of these genes, respectively (Fig. 3B). The results showed that CAS, FAS, IPAS, and AAS all resulted in two distinct and stable groups, with CAS divided into the C1 cluster (*n* = 10) and the C2 cluster (*n* = 19); FAS divided into the C3 cluster (*n* = 14) and the C4 cluster (*n* = 12); IPAS divided into the C5 cluster (*n* = 10) and the C6 cluster (*n* = 4); and AAS into the C7 cluster (*n* = 6) and the C8 cluster (*n* = 3) (Fig. 3C). The immune infiltration analysis showed that the C1 cluster of CAS had a higher abundance of resting memory CD4 T cells, activated NK cells, and neutrophils, while the C2 cluster had a higher abundance of γ-δ T cells and M0 macrophages; the C3 cluster of FAS had a higher abundance of plasma cells, resting memory CD4 T cells, and M1 macrophages, while the C4 cluster had a higher abundance of follicular helper T cells and M0 macrophages; the C6 cluster of IPAS had a higher abundance of resting mast cells; and the C8 cluster of AAS had a higher abundance of M2 macrophages (Fig. 3D).

The GSVA results showed that the C1, C3, and C7 clusters are mainly involved in various cardiomyopathies and cancers and amino acid metabolism-related pathways; the C2, C4, C5, and C8 clusters are mainly involved in various glycolipid metabolism, biosynthesis, metabolic processes, and immune disorders; whereas the C6 cluster is involved in cell adhesion-related pathways (Fig. 3E). These findings suggest that, based on macrophage polarization, AS samples from different regions can be distinguished into two subgroups with significantly different biological functions, particularly biosynthetic and metabolic processes, immune responses, and cardiomyopathies.

### WGCNA and screening of gene modules

**Figure 4:**
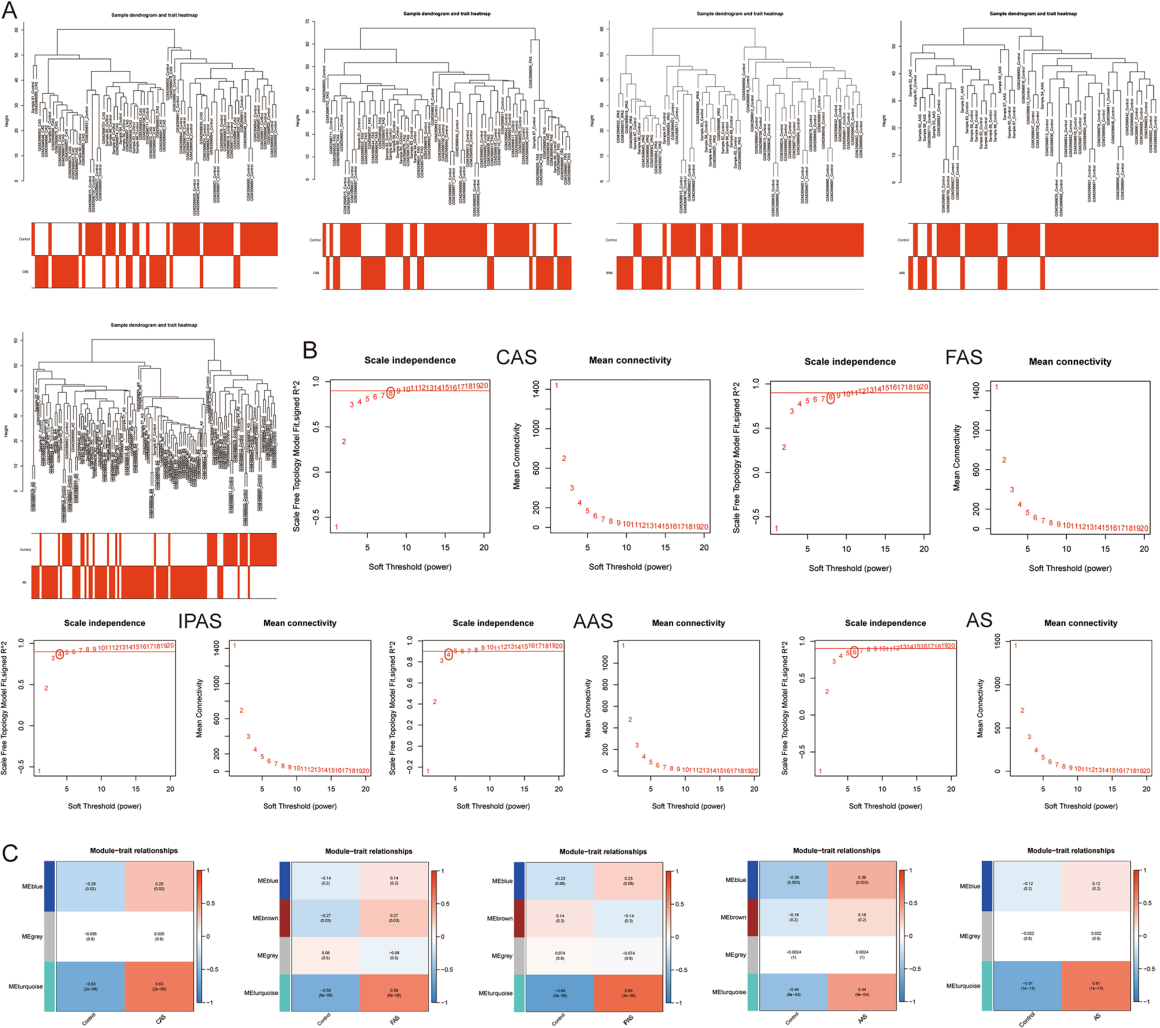
Co-expression network of differentially expressed genes. (**A**) The sample clustering plot after removing outlier samples; (**B**) The selection of soft threshold power; (**C**) Correlation analysis between module eigengenes and clinical status.

WGCNA was used to screen for co-expressed modules most relevant to AS and macrophage polarization. WGCNA was first performed on different regional AS samples and control samples to obtain the most relevant modules for AS. The results showed that 3, 4, 4, 4, and 3 co-expression modules with CAS, FAS, IPAS, AAS, and AS were obtained at soft thresholds of 8, 8, 4, 4, and 6, respectively. The “turquoise” module showed a significantly positive correlation with all of them; thus, 3671, 3728, 3873, 3187, and 815 module genes related to CAS, FAS, IPAS, AAS, and AS were obtained, respectively (Fig. 4A–C).

### Identification of Hub genes

**Figure 5:**
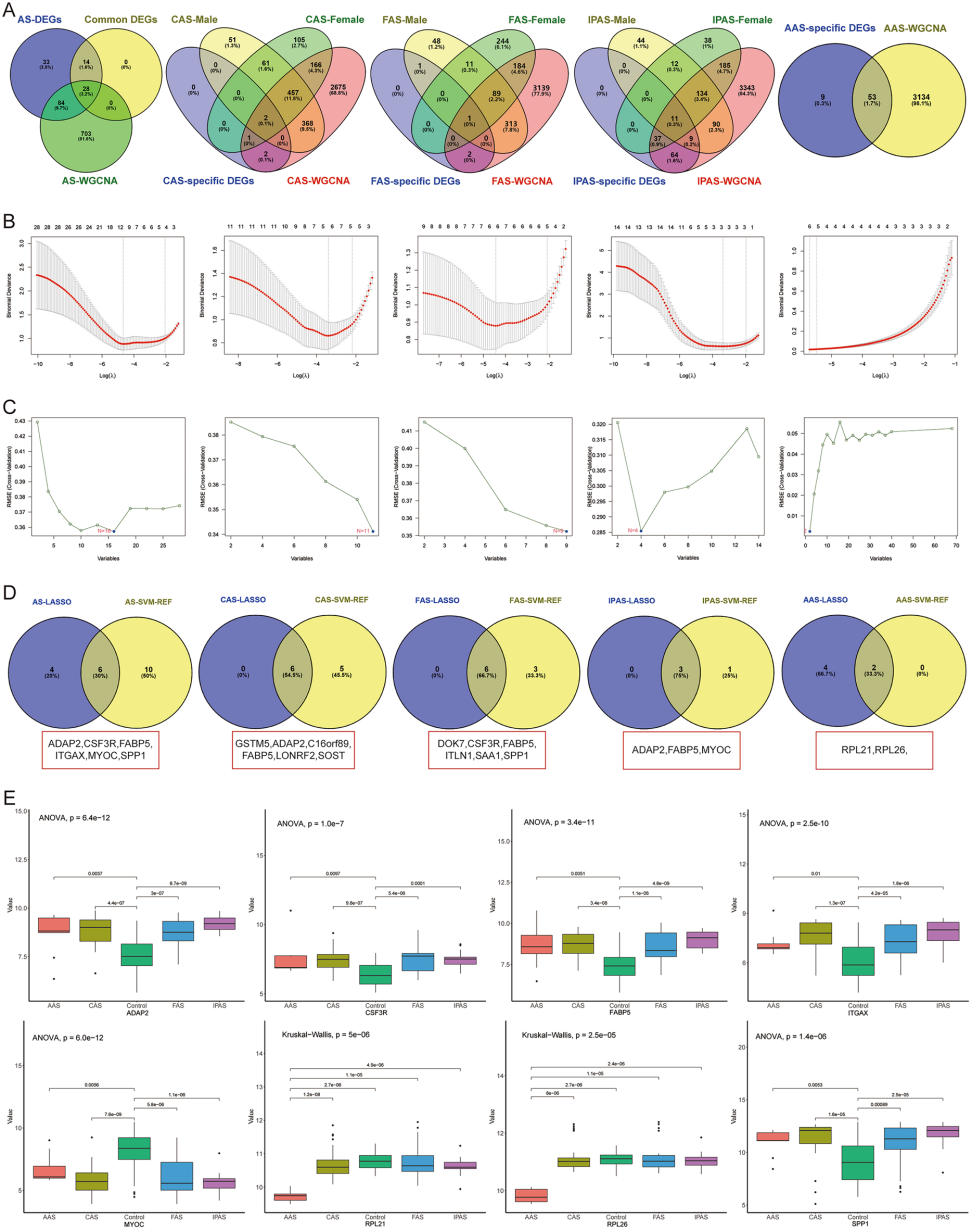
Machine learning modeling and single-gene differential analysis to identify Hub genes. (**A**) Venn diagram showing candidate Hub genes; (**B**) LASSO regression algorithm to screen feature genes; (**C**) SVM-REF to screen key genes; (**D**) Venn diagram showing potential Hub genes; (**E**) Single-gene differential analysis to screen for Hub genes.

First, AS-DEGs, module genes of AS, and common DEGs were intersected to obtain 28 candidate Hub genes for AS. Then the intersection of CAS, FAS, IPAS, and AAS-specific DEGs with module genes to obtain candidate Hub genes. In order to generalize the diagnostic efficacy of Hub genes, the samples were stratified by gender to explore whether there were differences in the expression of specific DEGs. The results showed that 939 DEGs for CAS, 463 DEGs for FAS, and 300 DEGs for IPAS were identified in males (*P* < 0.05 & |logFC| > 1.0), and 792 DEGs for CAS, 529 DEGs for FAS, and 417 DEGs for IPAS were identified in females (*P* < 0.05 & |logFC| > 1.0), and that 22, 1, 11, and 53 candidate Hub genes for CAS, FAS, IPAS, and AAS were identified by intersecting with the above genes, respectively (Fig. 5A). Therefore, Hub genes from different regions of AS and AS were used for subsequent screening.

To further screen Hub genes and reduce the bias of diagnostic models, LASSO regression and SVM-REF were used to screen the characterized key genes. The results showed that LASSO regression screened 10, 6, 6, 3, and 6 featured genes of AS, CAS, FAS, IPAS, and AAS (Fig. 5B); SVM-REF screened 16, 11, 9, 4, and 2 key genes of AS, CAS, FAS, IPAS, and AAS (Fig. 5C), and ultimately obtained 6, 6, 6, 3, and 2 Hub genes of AS (ADAP2, CSF3R, FABP5, ITGAX, MYOC, and SPP1), CAS (GSTM5, ADAP2, C16orf89, FABP5, LONRF2, and SOST), FAS (DOK7, CSF3R, FABP5, ITLN1, SAA1, and SPP1), IPAS (ADAP2, FABP5, and MYOC), and AAS (RPL21 and RPL26) (Fig. 5D).

Then, the expression differences of these genes among different AS were verified by single-gene differential analysis, which showed that ADAP2, CSF3R, FABP5, ITGAX, MYOC, and SPP1 of the AS samples were significantly different from the other AS; RPL21 and RPL26 of the AAS samples were significantly lower than those of the other groups (Fig. 5E). Thus, we obtained 6 Hub genes for AS (ADAP2, CSF3R, FABP5, ITGAX, MYOC, and SPP1) and 2 Hub genes for AAS (RPL21 and RPL26).

**Figure 6:**
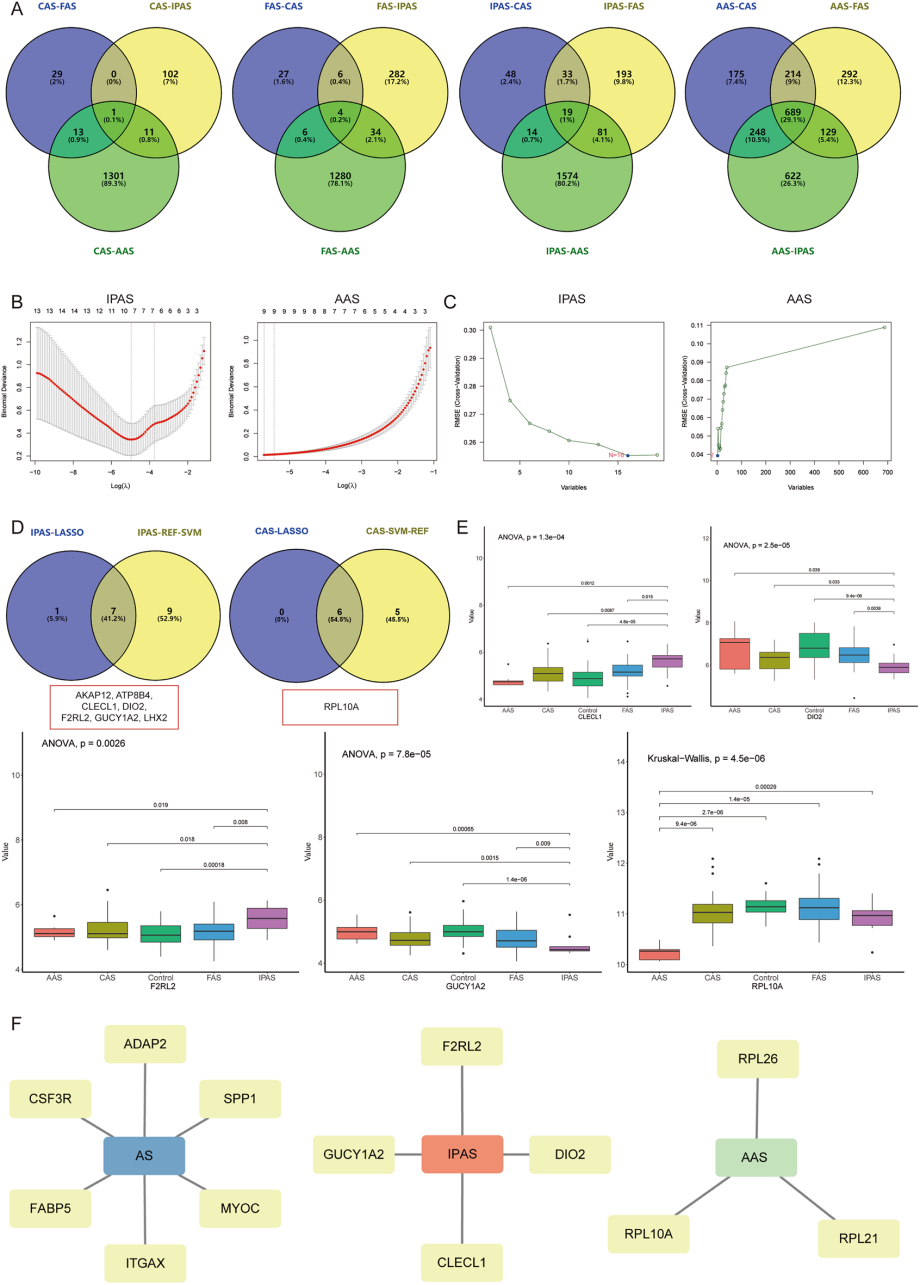
Identification of differential expression analysis between different arterial bed AS and screening of Hub genes. (**A**) Venn diagram showing the common specific DEGs of CAS, FAS, IPAS, and AAS; (**B**) LASSO regression algorithm to screen feature genes for IPAS and AAS; (**C**) SVM-REF to screen key genes for IPAS and AAS; (**D**) Venn diagram showing potential Hub genes for IPAS and AAS; (**E**) Single-gene differential analysis to screen for Hub genes; (**F**) Hub gene network diagram of different arterial beds AS.

In order to obtain more potential Hub genes, differential expression analysis was also performed on different AS samples, and 1, 4, 19, and 689 potential Hub genes (*P* < 0.05 & |logFC|> 0.25) were identified for CAS, FAS, IPAS, and AAS, respectively (Fig. 6A). To minimize overfitting, LASSO regression and SVM-REF were performed on the potential Hub genes of IPAS and AAS to obtain 6 potential Hub genes of IPAS and 6 potential Hub genes of AAS, respectively (Fig. 6B,D). Then, four Hub genes for IPAS (CLECL1, DIO2, F2RL2, and GUCY1A2) and one Hub gene for AAS (RPL10A) were obtained by single-gene difference analysis (Fig. 6E).

Finally, six Hub genes for AS (ADAP2, CSF3R, FABP5, ITGAX, MYOC, and SPP1) and four Hub genes for IPAS (CLECL1, DIO2, F2RL2, and GUCY1A2) and three Hub genes for AAS (RPL21, RPL26, and RPL10A) were obtained by differential expression analysis between AS and normal samples and between samples with different AS (Fig. 6F).

### Construction and evaluation of models

**Figure 7:**
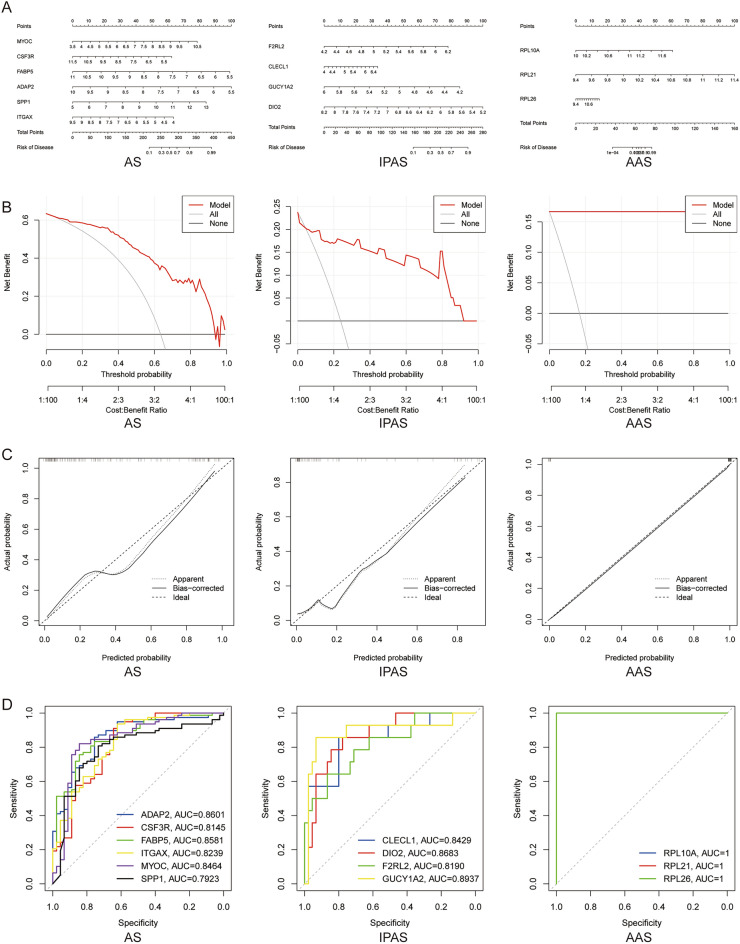
Construction and evaluation of nomogram. (**A**). Construction of a nomogram for predicting the risk of AS, IPAS, and AAS based on SVM model; (**B**, **C**). Construction of (**B**) calibration curve and (**C**) decision curve analysis for assessing the predictive efficiency of the nomogram model; (I). ROC curves predict the diagnostic efficacy of the Hub gene.

To further assess the predictive efficiency of the SVM model, a nomogram was constructed to estimate the risk of occurrence in AS and different regions of AS (Fig. 7A), and then the predictive efficiency of the nomogram was assessed using calibration curve and decision curve analysis (Fig. 7B,C). The ROC curves demonstrated that all of these genes had satisfactory diagnostic efficacy in distinguishing between normal and AS (ADAP2, AUC = 0.8601; CSF3R, AUC = 0.8145; FABP5, AUC = 0.8581; ITGAX, AUC = 0.8239; MYOC, AUC = 0.8464; SPP1, AUC = 0.7923), IPAS (CLECL1, AUC = 0.8429; DIO2, AUC = 0.8683; F2RL2, AUC = 0.8190; GUCY1A2, AUC = 0.8937), and AAS (RPL21, AUC = 1; RPL26, AUC = 1; RPL10A, AUC = 1) samples from different regions (Fig. 7D).

## Discussion

AS commonly occurs in systemic arteries and exhibits certain similarities across different arterial beds. In our study, we initially conducted a differential expression analysis comparing AS samples with control samples, and the results demonstrated significant associations between AS pathogenesis and inflammatory response, cell migration and chemotaxis, cell death, lipid metabolism, and immune response (Fig. 2C), all of which were confirmed^25^. Subsequently, six hub genes for AS were identified, which effectively distinguished between normal and AS samples, among which ADAP2, CSF3R, FABP5, ITGAX, and SPP1 were highly expressed in AS, whereas MYOC expression was downregulated (Fig. 5E).

ADAP2 encodes ArfGAP with the double PH domain 2 protein, which plays an important role in cardiac development. Cardiovascular malformations caused by patients with NF1 microdeletions syndrome are associated with ADAP2 dysfunction^26^, and its high expression is seen in patients with myocardial infarction^27^.

CSF3R is essential for granulocyte maturation and is primarily involved in the regulation of inflammation. The expression of colony-stimulating factor and its receptor is up-regulated in patients with multiple stages of acute myocardial infarction and stable angina pectoris, suggesting that adhesion, chemotaxis, and phagocytosis of neutrophils and monocyte macrophages are enhanced with the development of AS and acute myocardial infarction^28^. In addition, the CSF3/CSF3R axis is involved in lipid metabolism and promotes the development of nonalcoholic fatty liver disease and AS^29,30^.

FABP5 plays a role in the transportation, regulation, and metabolism of lipids. Its high expression in AS has been demonstrated, which is not only considered a potential biomarker for residual risk of AS but also a sensitive marker for lipid-rich macrophages on the luminal side of AS lesions^31–33^.

ITGAX is involved in early AS lesion formation and is highly expressed in AS tissues, and several bioinformatic analyses have suggested it as a potential biomarker for AS^34,35^. The mice with double knockouts of ITGAX and Apoe demonstrated lower monocyte adhesion and AS plaque macrophage content^36^. ITGAX was found to promote AS progression by regulating B differentiation through the TNF-α signaling pathway^37^.

MYOC is closely associated with glaucoma and has high expression levels in skeletal muscle and hearts in addition to the eye^38^. However, the relationship between MYOC and AS remains unclear, and its secreted glycoproteins may influence AS by regulating processes such as cell adhesion, cytoskeleton, and migration^39,40^.

SPP1 exhibits high expression in endothelial cells, macrophages, and vascular smooth muscle cells present in atherosclerotic plaques^41,42^. The level of SPP1 expression is closely associated with the development and severity of coronary artery disease^43^. Additionally, it has been identified as a potential marker for monitoring the severity of AS and predicting cardiovascular event-related mortality^44^.

Currently, there are several serum markers used to identify early AS, such as lipoproteins, ceramides, C-reactive proteins, high-sensitivity C-reactive proteins, matrix metalloproteinases, and miRNAs, among which high-sensitivity C-reactive proteins are comprehensively validated in the prediction of atherosclerotic cardiovascular events^45–47^. In our study, we identified six Hub genes for AS, and the nomogram demonstrated satisfactory diagnostic efficacy. These genes may supplement the diagnostic biomarkers for early AS.

Then, we conducted differential expression analysis on different types of AS and control groups to obtain DEGs and specific DEGs, and enrichment analyses showed that CAS was related to cellular differentiation, calcium and potassium homeostasis; FAS with cellular chemotaxis; IPAS with osteoclast differentiation and regulation of interleukin factors; and AAS with inflammation and cell migration (Fig. 2D–F). Previous studies reported higher foam cell lesions and lipid plaques in CAS and higher calcification and lower cholesterol concentrations in FAS^8,48^. In our study, we found that CAS is involved in the regulation of pathways related to calcium homeostasis, which may explain why CAS has a lower degree of calcification.

In addition, we identified four Hub genes for IPAS (CLECL1, DIO2, F2RL2, and GUCY1A2) and three Hub genes for AAS (RPL21, RPL26, and RPL10A) after high-intensity screening. The CLECL1 and F2RL2 expressions were significantly up-regulated in IPAS compared to control and other types of AS, while DIO2 and GUCY1A2 expressions were down-regulated; the RPL21, RPL26, and RPL10A expressions were significantly down-regulated in AAS compared to control and other AS.

CLECL1 plays an important role in the regulation of the immune response, inducing phosphorylation of p44/42 and JNK MAPK as well as up-regulation of MHC class II expression, resulting in a partially mature dendritic cell phenotype^49^; it also serves as a T-cell co-stimulatory molecule to bias the Th2 response by enhancing IL-4 secretion from CD4(+) T cells^50^.

F2RL2 mediates thrombin-induced proliferation, migration, and matrix biosynthesis, and the production of inflammatory and pro-growth mediators, which contribute to the progression of inflammatory diseases^51,52^.

DOI2 is mainly present in brown adipose tissue, placenta, pituitary and muscle and is responsible for deiodinating T4 to T3^53^. The upregulation of DIO2 gene expression was observed in ApoE mice with hyperlipidemia and AS in Western diets, indicating that DIO2 plays a certain role in the development of AS^54^.

GUCY1A2 is the active subunit of guanylate cyclase, and deficiency of guanylate cyclase function contributes to the formation of AS plaques in LDL^-/-^ mice, which may be associated with increased adhesion of leukocytes to the endothelium^55,56^. GUCY1A2 also protects against cerebral ischemia perfusion injury by mitigating apoptosis and mitochondrial damage^57^.

RPL21, RPL26, and RPL10A are important components of the large 60S ribosomal subunit with functions involved in cell proliferation, differentiation, apoptosis, and DNA repair, which have been associated with metabolism and cardiovascular disease progression^58^. However, the mechanism of its specific involvement in AAS remains undefined, with some studies suggesting a possible association with inflammation and mitochondrial apoptosis^59^.

The immune infiltration analysis revealed significant differences between macrophages and mast cells between AS and control samples. Therefore, we concluded that macrophage polarization plays an important role in the progression of AS. We then classified AS samples based on macrophage polarization-related genes and obtained different molecular clusters, which showed significant biofunctional differences between biosynthesis metabolism and cardiomyopathy. M1 macrophages produce pro-inflammatory factors that are involved in the clearance of pathogens, while M2 macrophages produce anti-inflammatory factors that promote tissue repair and fibrosis. In the early stages, M2 macrophages are predominantly present in the plaques and stabilize them. As the lesions progress, the number of M2 macrophages decreases, the number of M1 macrophages gradually increases, and the secretion of pro-inflammatory factors increases, which promotes the development of AS and renders the plaques susceptible to rupture^60^. In addition, we found that AAS has a lower abundance of M1 macrophages, which may be related to the involvement of anti-inflammatory-related pathways (Th1 and Th2 cell differentiation, Th17 cell differentiation, IL-17 signaling pathway).

We found that AS has a higher abundance of activated mast cells, which were found to promote AS and plaque development by stimulating endothelial cells to express adhesion molecules, recruiting neutrophils and circulating leukocytes to plaques, secreting tryptase to disrupt endothelial integrity, and secreting histamine to alter microvascular permeability^61^.

To summarize, we obtained DEGs and specific DEGs by differential expression analysis among different samples, and then, after screening by multiple methods of gender stratification, key module genes, machine learning models, and single-gene differential analysis, we finally identified 6 Hub genes for AS, 4 Hub genes for IPAS, and 3 Hub genes for AAS. Immune infiltration analysis revealed that AS had a higher abundance of M0 macrophages and activated mast cells, while AAS had a lower abundance of M1 macrophages. Functional enrichment analysis indicated that CAS was involved in the regulation of calcium homeostasis; IPAS was involved in ossification; and AAS was involved in inflammation-related pathways.

However, there are still some limitations to our study. First, we did not investigate other large arteries such as cerebral arteries, coronary arteries, and thoracic aortas due to missing datasets, and we were unable to merge more datasets for our study. Secondly, we did not perform prognostic analysis. AS as a non-oncologic disease, the GEO database seldom contains the basic information and prognostic information of the patients. Due to the disease selection and database limitations, we were unable to perform the follow-up study for prognostic analysis. Finally, we identified the Hub genes of IPAS and AAS, which show satisfactory diagnostic efficacy, but the specific mechanisms of Hub genes in IPAS and AAS remain unclear.

## Conclusions

There are similarities and heterogeneities in the pathogenesis of AS between different arterial beds.

### Supplementary Information

Supplementary Information 1.

Supplementary Information 2.

## Data Availability

The following information was supplied regarding data availability: Data is available at NCBI GEO: GSE100927 and GSE57691. Additionally, any analytic technology-related questions can be directly contacted by the corresponding author.

## Abbreviations

AS: Atherosclerosis
AAS: Abdominal aortic atherosclerosis
AUC: Area under curve
CAS: Carotid Atherosclerosis
DEGs: Differentially expressed genes
FAS: Femoral atherosclerosis
GEO: Gene Expression Omnibus Database
GO: Gene ontology
GSEA: Gene set enrichment analysis
GSVA: Gene set variation analysis
KEGG: Kyoto Encyclopedia of Genes and Genomes
IPAS: Infrapopliteal Atherosclerosis
LASSO: Least Absolute Shrinkage and Selection Operator Regression
ROC: Receiver Operating Characteristic
SVM-RFE: Support Vector Machine-Recursive Feature Elimination
WGCNA: Weighted gene co-expression network analysis
